# Novel *WDR26* variant in a Chinese patient with Skraban-Deardorff syndrome: a case report and literature review

**DOI:** 10.3389/fped.2026.1897123

**Published:** 2026-07-15

**Authors:** Cuiyun Li, Ying Xu, Guiying Zhang, Liting Chen, Hui Zeng, Wei Li, Ying Yu

**Affiliations:** Department of Medical Genetics and Antenatal Diagnostic Center, Hainan Branch, Shanghai Children's Medical Center, School of Medicine, Shanghai Jiao Tong University, Sanya, China

**Keywords:** Chinese, delayed language development, epilepsy, Skraban-Deardorff syndrome, WDR26

## Abstract

*De novo* variants of the *WDR26* gene leading to haploinsufficiency have recently been associated with Skraban-Deardorff syndrome. The syndrome is an extremely rare autosomal dominant neurodevelopmental disorder that exhibits a wide range of clinical features including intellectual disability, delays in development, seizures, unusual facial characteristics, weak muscle tone, abnormal walking pattern, and multiple structural abnormalities. Here, we report a Chinese pediatric case of Skraban-Deardorff syndrome, wherein genetic testing revealed a novel *de novo*, heterozygous frameshift variant c.271delA (p.Thr91Profs*40) of the *WDR26* gene. By reviewing previously reported cases, we found that dysfunction of the *WDR26* gene does not necessarily accompany the occurrence of seizures. To date, epilepsy has not been a major symptom in any of the reported cases in China. Instead, delayed language development should be considered as the typical clinical phenotype of Skraban-Deardorff syndrome. It is worth noting that while epilepsy can be managed by medication, delayed language development does not have a straightforward treatment. If timely and adequate language interventional therapy and alternative communication methods are not implemented, the prognosis of children with Skraban-Deardorff syndrome may be significantly compromised.

## 1 Introduction

**Figure 1:**
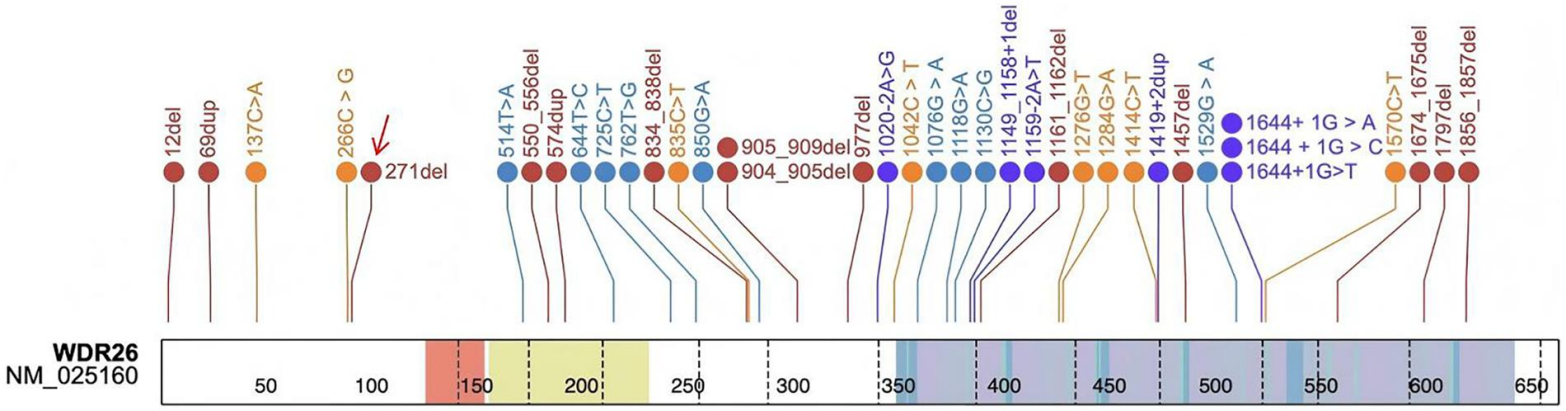
Schematic of the *WDR26* gene and WDR26 protein with position of variants from previously published and a novel *WDR26* variant identified in SKDEAS is indicated. The novel *WDR26* variant identified in this study (c.271delA) is indicated by the red arrow. The orange box corresponds to the LisH domain, and the yellow box corresponds to the CTLH domain. The blue and purple boxes correspond to the WD40 domains and WD40 repeats, respectively. Missense variants are marked in blue line, frameshift variants, nonsense variants and splicing variants are marked in red, orange and purple lines.

Skraban-Deardorff syndrome is a rare autosomal dominant neurodevelopmental disorder caused by *WDR26* gene variants (1). *WDR26* is located on chromosome 1q42.12, spanning across 14 exons. It has been documented that there is a resemblance between the clinical characteristics of people with *WDR26 de novo* mutations and those with 1q41q42 microdeletion, with the insufficient activity of the *WDR26* gene being identified as the underlying mechanism (2–4). To date, 36 pathogenic variants of the *WDR26* gene have been reported, with three cases identified through whole exome sequencing (WES) but lacking a detailed clinical description (1, 4–15). The reported variants comprise nine missense variants, thirteen frameshift variants, eight nonsense variants, and six splicing variants (Figure 1). Clinical symptoms of Skraban-Deardorff syndrome are heterogeneous and cover a broad spectrum of symptoms ranging from neurologic manifestations to non-neurologic ones. The most frequently reported phenotypes include intellectual disability (ID), developmental delay (DD), hypotonia, seizures, gait abnormality, facial feature abnormalities, and multiple structural anomalies (1, 8). Owing to the limited number of reported cases, detailed descriptions of the clinical phenotypes require further confirmation and delineation, especially in the Chinese population. To date, 25 patients have been reported in Europe and the United States (1, 4, 5, 11, 12), whereas only 8 cases have been documented in China (9, 10, 13–15). Here, we report a new case of Skraban-Deardorff syndrome with a novel heterozygous variant of the *WDR26* gene. Furthermore, we provide clinical and molecular data and compare the phenotypes of the Chinese probands with the data in the reported literature.

## 2 Case presentation

**Figure 2:**
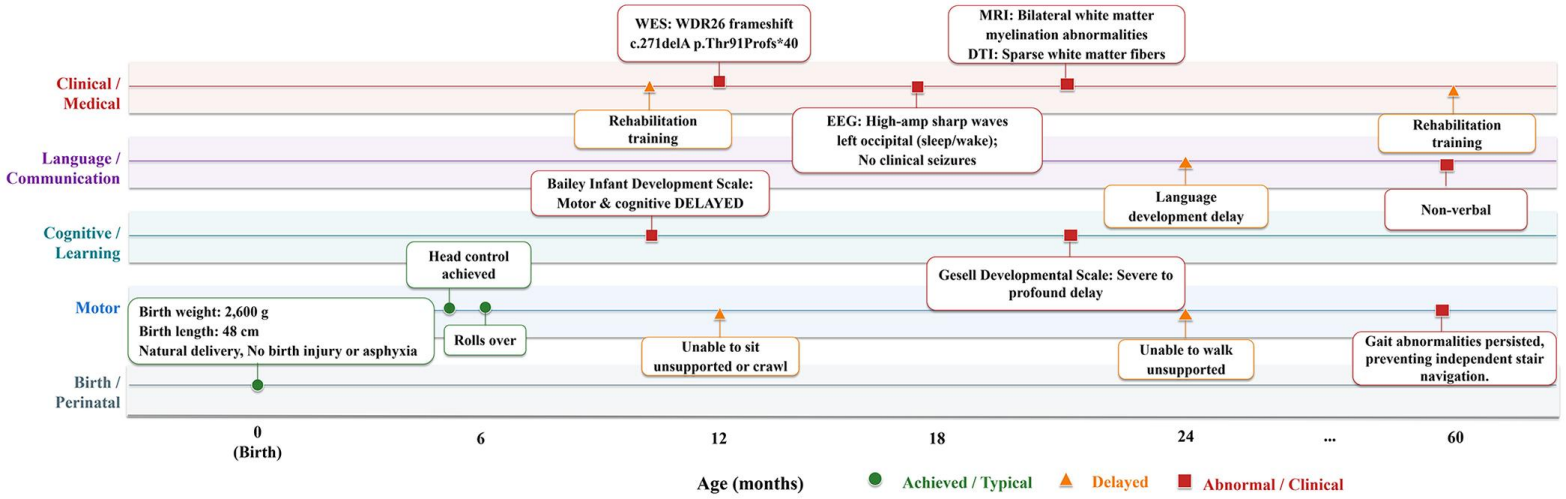
Clinical timeline of the patient according to CARE guidelines (0–5 years). This figure summarizes the chronological progression from birth to 5 years of age, detailing key developmental milestones, diagnostic assessments (WES, MRI, EEG), and therapeutic interventions. Green circles indicate achieved milestones; orange triangles indicate delayed milestones; red squares indicate abnormal findings.

The patient was a 2-year-old boy, the only child of non-consanguineous Han Chinese parents. There were no notable antenatal issues in his medical history. An ultrasound of the mother at the 30-week prenatal examination showed a small fetus. There were no medical interventions, and the mother of the patient gave birth at 37 weeks of gestation through natural labor. There was no birth trauma or asphyxiation. The patient's birth weight was 2,600 g, and length was 48 cm. Difficulties with feeding were noted. Both his parents were thalassemia carriers, and his thalassemia test results showed intermediate thalassemia as -ɑ3.7 and --SEA composite heterozygous carrier. His hemoglobin concentration (HGB) was 95 g/L, which presented as mild anemia. His parents were in good health and had no hereditary disease history. The clinical timeline of the patient is illustrated in Figure 2.

**Figure 3:**
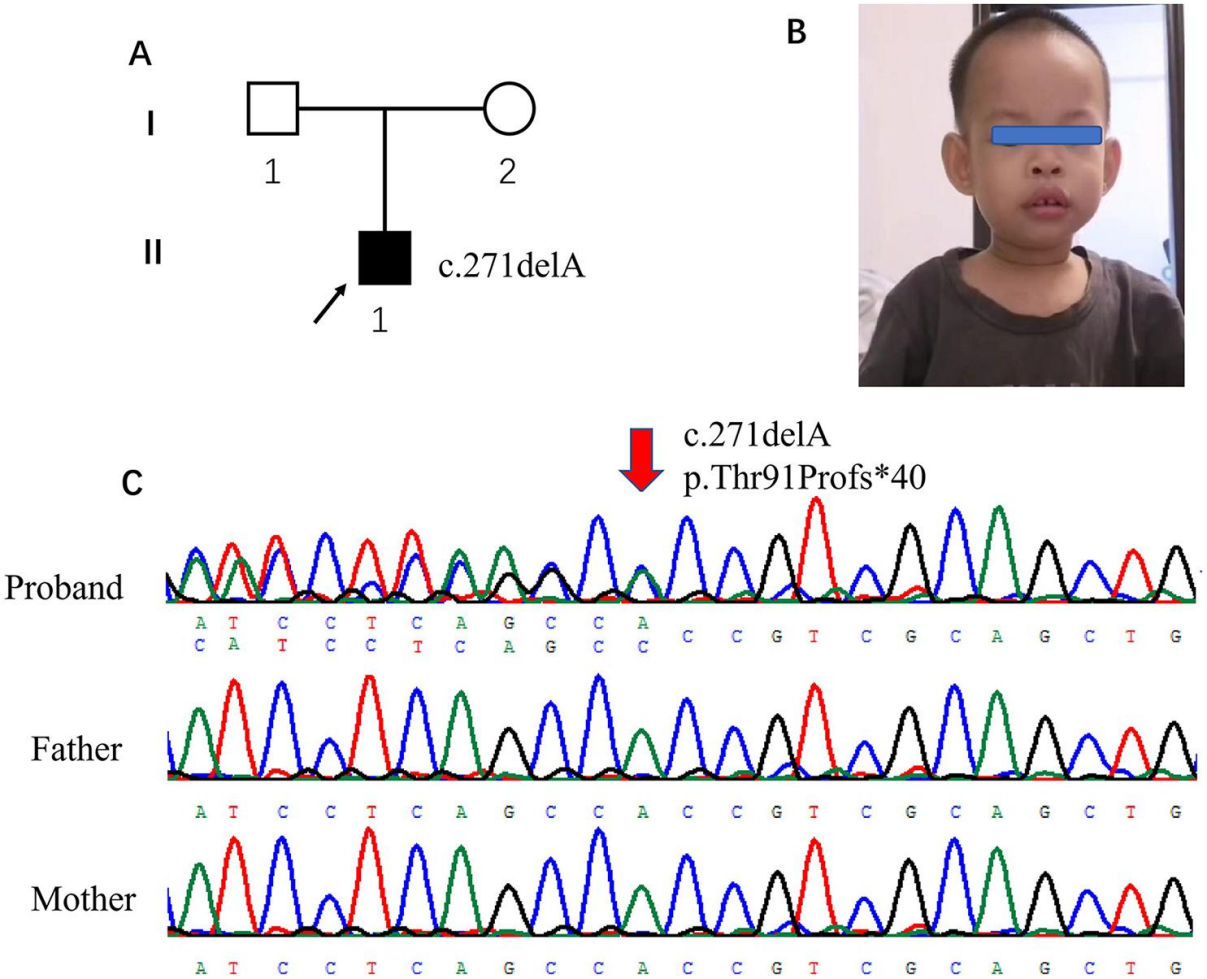
Clinical phenotype and gene result. **(A)** Family pedigree of this case. **(B)** Distinctive facial features were identified. **(C)** Sanger sequencing of the variant in the pedigree and location of the variant in the gene. Sanger sequencing results for the c.271delA variant in case 1. The parents were variant negative.

The patient began exhibiting signs of developmental delay 4 months after birth. At 5 months, he was able to look up, and by 6 months, he could roll over, but he still needed assistance to sit upright. The patient's parents first sought medical help for developmental delay at Sanya Women and Children's Hospital managed by Shanghai Children's Medical Center, when the child was 8 months old. Physical examination showed that he had a height of 68 cm (P10–20), weight of 6 kg (<P3), and head circumference of 41 cm (<P3). His cognitive and motor development was assessed using the Bailey Criteria for Infant and Child Development, revealing significantly delayed development in both motor skills and cognition (Motor Scale Developmental index < 50; Intelligence Scale Developmental Index = 50). This assessment suggested that the patient had a mental age equivalent to 4 months. No other abnormalities were found, including those of ear, nose, throat, eyes, and respiratory, musculoskeletal, and cardiac systems. Distinctive facial features were observed, including a protruding forehead, depressed nasal root, full nasal tip, full lips, wide mouth, widely spaced teeth, enamel hypoplasia, and abnormal gums (Figure 3B). Hypotonia was also detected. Prior to the availability of whole exome sequencing (WES) results, we conducted a comprehensive differential diagnosis based on the patient's clinical presentation. Given the developmental delay, hypotonia, and dysmorphic facial features, 1q41q42 microdeletion syndrome was initially considered, as the *WDR26* gene is located within the 1q42.12 region. Additionally, common genetic causes of syndromic developmental delay, such as Fragile X syndrome and chromosomal aneuploidies, were clinically excluded based on G-banding karyotyping and targeted molecular testing. Subsequently, the patient underwent comprehensive rehabilitation training, including physical therapy (twice weekly) focusing on gross motor control and speech therapy (twice weekly) targeting language stimulation. The patient showed good tolerability to the interventions, though progress remained slow.

**Figure 4:**
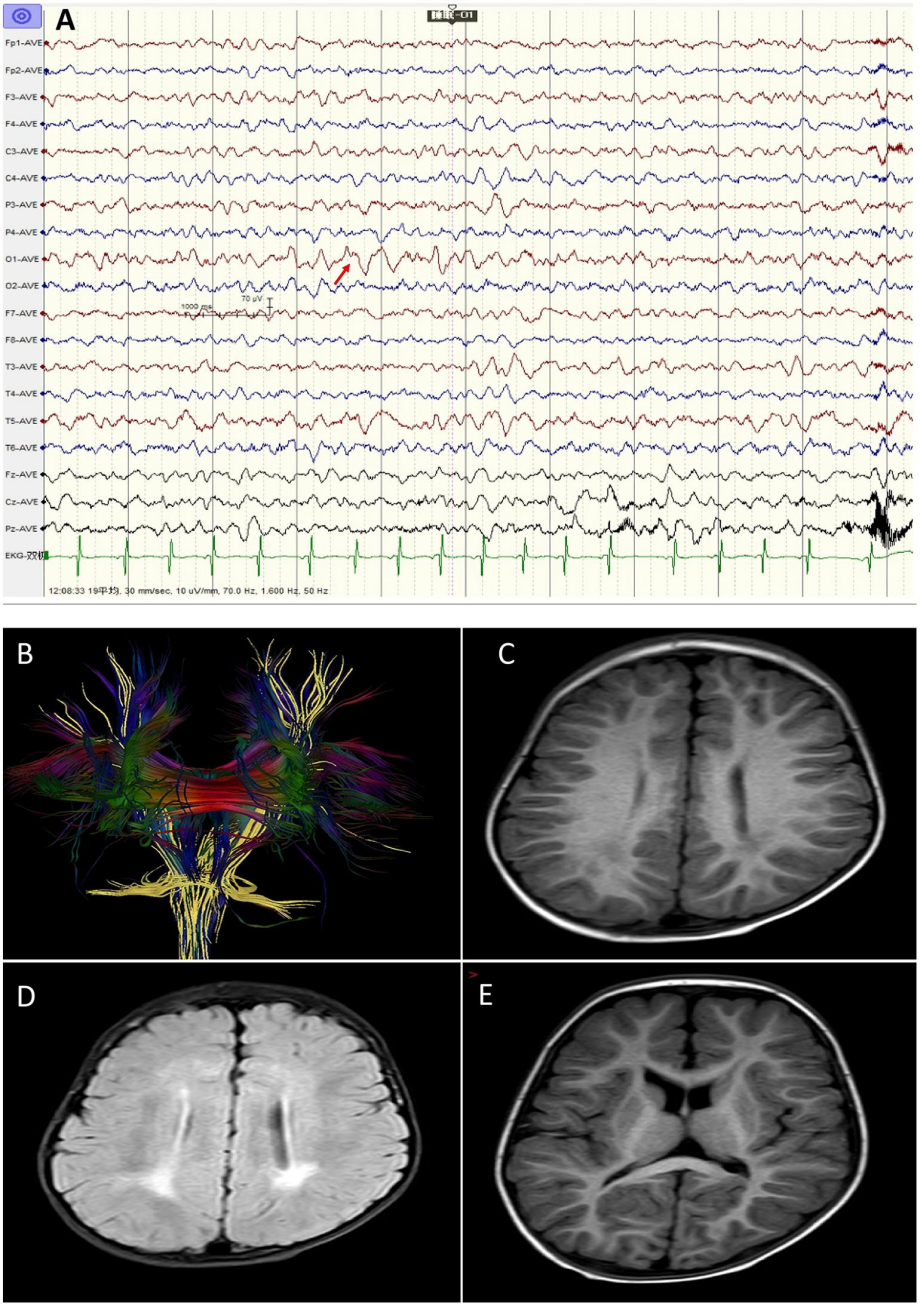
Neuroimaging and electroencephalographic findings **(A)** electroencephalography (EEG) showing focal epileptiform discharges (indicated by the red arrow) originating from the right occipital lobe. **(B)** DTI demonstrating sparse bilateral white matter tracts. **(C–E)** Axial MRI showing delayed myelination of the bilateral cerebral white matter, predominantly in the periventricular regions.

By the age of 12 months, the patient was still unable to sit without support or crawl. Despite the absence of any previously detected seizures or epileptic activity, an electroencephalogram conducted at 18 months of age revealed the presence of a few occasional low-high sharp and sharp-slow waves in the left occipital area during both sleep and wakefulness (Figure 4A). Additionally, follow-up magnetic resonance imaging (MRI) at 21 months of age revealed abnormal bilateral white matter myelination, especially in the paraventricular white matter. Meanwhile, diffusion tensor imaging (DTI) showed sparse white matter fiber tracts in the bilateral cerebral hemispheres (Figures 4B–E).

Neurodevelopmental status was evaluated using Gesell Developmental Schedules at 21 months of age. These assessment results showed that the developmental quotient (DQ) of his four domains fell within the scope of severe to extremely severe lag behind, including scores of 31, 32, 30, 23, and 21 for adaption, gross motor, fine motor, language, and personal/social functions, respectively. Despite receiving specialized training since 8 months of age, he was still unable to walk without support at the age of 24 months. His cognitive development was severely delayed, manifesting as aphasia. Medical examination showed that he had a height of 86 cm (<P3) and weight of 8 kg (<P3). A follow-up visit at approximately 5 years of age revealed persistent severe impairments. The patient remained non-verbal and could not utter even simple words such as “baba” or “mama”. He was not toilet-trained and required assistance for daily living activities. Although he could comprehend simple instructions, he exhibited poor compliance and was often uncooperative during interactions. Additionally, he presented with an abnormal gait and was unable to ascend or descend stairs independently. Currently, he does not attend school and undergoes comprehensive rehabilitation training five days a week; however, the therapeutic effect remains minimal.

## 3 Materials and methods

### 3.1 Whole exon sequencing (WES)

Peripheral blood samples were collected at the Sanya Women and Children's Hospital managed by Shanghai Children's Medical Center. Following the manufacturer's protocol of the Gentra Puregene Blood Kit (Magen, China), genomic DNA from the patient and his parents were respectively extracted from their peripheral blood samples. Next, whole-exome capture was performed using the Agilent SureSelect V6 enrichment capture kit (Agilent Technologies, Inc., Woburn, MA, USA). Sequencing of the captured library was carried out using the Illumina HiSeq 2,500 System (Illumina, Inc., San Diego, CA, USA). FastQC (version 0.11.2) was used as the quality control standard to evaluate all the raw sequencing data. As previously reported (16), GATK software was used to detect variations in the bam files that passed quality control and to generate VCF format files. The mutations were put through screening and annotation using the EGIS online software. First, the common benign mutations and those outside the capture area were removed. Then, the genes were screened according to the degree of phenotypic conformity, the variant genes were filtered according to the nature of variation, and the candidate sites of pathogenic variants were identified. Finally, the pathogenic genes were identified through family verification and ACMG variation classification. The variants detected using next-generation sequencing were subsequently confirmed using Sanger sequencing.

### 3.2 Literature review

We search the re1evant literatures about *WDR26* variants in patients with Skraban-Deardorff syndrome, using the keywords “Skraban Deardorff” or “SKDEAS” or “WDR26” in the PubMed database and the China National Knowledge Infrastructure (CNKI) database and Web of Science and Embase (from inception until November 2025), and summarize the results. Duplicate reports of the same patient were excluded. Data extraction was performed independently by two authors (C.L. and Y.X.), and discrepancies were resolved by consensus with a senior author (W.L.).

## 4 Results

Whole-exome sequencing revealed a novel frame-shift variant, c.271delA, p.Thr91Profs*40, in the sixth exon of *WDR26* (NM_025160.6) in the patient. This variant involves the deletion of a single adenine base at position 271 (c.271delA), resulting in a frameshift mutation. It causes the 91st amino acid to change from threonine (Thr) to proline (Pro) and shifts the reading frame. Under this new frame, translation continues for approximately 40 amino acids after position 91 until a stop codon is encountered (p.Thr91Profs*40), leading to a truncated protein with severely impaired or complete loss of function (PVS1). This frameshift variant is predicted to trigger nonsense-mediated mRNA decay (NMD), which supports the proposed loss-of-function mechanism via haploinsufficiency. Sanger sequencing validated this *WDR26* variant as well as the wild-type status of his parents (PS2) (Figure 3A,C). This variation was not included in the human gene mutation database (HGMD) and the gene panel aggregation database (gnomAD) and other databases (PM2). Based on the ACMG guidelines of variant interpretation, this newly identified frame-shift variant was determined to be pathogenic (PSV1 + PS2 + PM2).

Through a detailed review of the literature, it was concluded that ten relevant studies comprising 37 patients with *WDR26* variants were identified and further analyzed, including our study one case (Table 1) (1, 4, 5, 9–15). However, there are 3 cases only reported the *WDR26* variation site, no clinical phenotype information was provided. To date, only nine Chinese cases have been reported, including the current case. Among them, there were 15 female cases (44.12%), 19 male cases (55.88%), 31 pediatric cases, and 3 adult cases. A total of 34 variant sites were identified, among which 1,644 + 1 shared different base variations at the same site, while the other variant sites were distinct. There were 13 frameshift variants, 9 nonsense variants, 6 missense variants, and 6 splice site variations, all of which were novel variants. 100% (34/34) of the cases had developmental delay and/or intellectual disability; 100% (34/34) had language impairment; 82.4% (28/34) had seizures, and 52.2%(12/23) with EEG abnormalities; 75.8% (25/33) had CNS structure abnormalities (abnormal ventricular size, abnormal periventricular white matter signal, pineal cyst, thinned corpus callosum, etc.); 67.6% (23/34) had hypotonia or hyperkinesia; 68.8% (22/32) had abnormal gait, such as wide-based gait and/or spastic gait; 78.3% (18/32) had a behavioral and/or psychiatric issues; 75.8% (25/33) had facial deformities, including rough facial skin, nasal hypoplasia and dental hypoplasia; 50% (15/30) had ophthalmic abnormalities; 23.5% (4/17) had hypoplastic nails; 27.3% (6/22) had gastrointestinal dysfunction; 50% (15/30) hadskeletal system abnormalities.

**Table 1:** Clinical features for patients with *WDR26*-related SKDEAS.

Clinical features	Foreign cases					Chinese cases						Total	Proportion (Chinese)
	Skraben et al., 2017	Cospain et al., 2021	Pavinato et al., 2021	Innella et al., 2022	Gunasekaran et al., 2022	Hu J et al., 2022	Shirley et al., 2022	Yang Q et al., 2024	ZY Han et al., 2025	Lin et al., 2025	Our study		
Sex(M/F)	5F10M	3F3M	2M	1M	1F	1F1M	1F	2F	1F	2M	1F	15F19M	6F3M
Age	24m-34y	18m-16y	8y/19y	18y	8y	3y/11y	12y	1y6m/ 11y	4m	2y11m/5y1m	2y	4m-34y	4m-12y
Developmental delay/intellectual disability	15/15	6/6	2/2	+	+	2/2	+	2/2	+	2/2	+	34/34	9/9
Language impairment	15/15	6/6	2/2	+	+	2/2	+	2/2	+	2/2	+	34/34	9/9
Seizures	15/15	5/6	2/2	-	+	0/2	-	1/2	+	2/2	-	28/34	4/9
EEG abnormality	6/10	0/1	0/1	-	+	0/2	-	1/2	+	2/2	+	12/23	5/9
CNS structure abnormalities	10/14	4/6	1/2	+	+	2/2	-	0/2	-	2/2	+	22/33	5/9
Hypotonia/Hyperkinesia	9/12	4/6	2/2	-	+	2/2	+	2/2	-	1/2	+	23/34	7/9
Abnormal gait	9/15	3/6	2/2	-	NA	2/2	+	2/2	+	2/2	NA	22/32	8/8
Behavioral and/or psychiatric issues	5/9	5/6	1/2	+	NA	1/2	-	2/2	+	2/2	NA	18/32	6/8
Facial deformities	12/15	2/6	1/2	+	NA	2/2	+	2/2	+	2/2	+	25/33	9/9
Ophthalmic abnormalities	9/14	1/6	NA	+	+	1/2	+	0/2	NA	1/2	NA	15/30	3/7
Hypoplastic nails	3/13	NA	NA	NA	NA	0/2	NA	1/2	NA	NA	NA	4/17	1/4
Gastrointestinal dysfunction	5/15	NA	0/2	NA	NA	0/2	NA	1/2	NA	0/1	NA	6/22	1/5
Skeletal system abnormalities	5/15	4/6	2/2	+	NA	0/2	+	2/2	NA	1/1	NA	15/30	4/7

## 5 Discussion

Skraban-Deardorff syndrome is an uncommon disease, which is characterized by DD, ID, epilepsy, abnormal gait, and recognizable facial dysmorphic features. The frequency of *WDR26* variants is estimated to be approximately 1 in 1,500 among individuals presenting with ID in European and American families, and approximately 1 in 2,000 among individuals exhibiting all phenotypes (1). However, its prevalence is still unknown in Asian patients, as variants have been uncommonly reported in Asians (9). These reported cases exhibit a wide spectrum of phenotypes. All patients experienced different degrees of developmental delay and/or intellectual disability. Here, we have reported a pediatric case of Skraban-Deardorff syndrome caused by a novel *de novo*, heterozygous frameshift variant c.271delA, p.(Thr91Profs*40) of the *WDR26* gene, along with detailed clinical information, including growth and observed development delays. The *de novo* frameshift variant detected in our case also results in haploinsufficiency of *WDR26*, which has been suggested as the pathogenic mechanism. *WDR26* is universally transcribed, exhibiting varying expression levels across various body tissues. Notably, its expression is higher in skeletal muscle, brain, liver, lung, and heart during fetal development, while in adults, it is primarily elevated in skeletal muscle and heart (1, 17, 18). The widespread prevalence of its expression could potentially account for the range of symptoms displayed by the disease.

According to previous studies, early-onset seizure is considered a stereotypical symptom of Skraban-Deardorff syndrome, with the age of onset ranging from the neonatal period to 7 years (1, 9, 10). In 2017, Skraban et al. first reported the discovery of *WDR26* gene variants in 15 patients with seizures. 82.4% (28/34) had seizures in the reported patients, while only 44.4% (4/9) had seizures in Chinese patients. Therefore, it may be concluded that dysfunction of *WDR26* is not always associated with seizures. On reviewing cases of Skraban-Deardorff syndrome reported in the literature, we found that all patients had delayed speech, and the severity could be significant. One patient had absent speech at 8 years of age, and the oldest individual in the cohort had dysarthric speech as an adult (1). In another report of a Chinese case, the patient could only speak five to six words and had difficulty understanding speech at the age of 11 years (9). In the presently reported case, he exhibited severe developmental delay in adaptation, gross motor and fine motor skills, and extremely severe developmental delay in language and personal/social functions. Severe language delay was already present at the age of 5 years, with the absence of word utterance. Consequently, delayed language development might be considered a core symptom of Skraban-Deardorff syndrome. Adequate and timely language therapy and augmentative/alternative communication methods could contribute to a better prognosis in children with Skraban-Deardorff syndrome. Such management can improve language development and reduce behavioral disorders by promoting communication and social skills (19, 20).

WDR26 is rich in protein-protein interaction domains (WDRs) and plays a significant role in various biological processes. *WDR26* is an evolutionary conserved gene and is highly intolerant to loss-of-function variation. This suggests haploinsufficiency as the underlying mechanism. However, the exact pathogenic mechanisms are still unclear, although the involvement of MAPK, Wnt, and PI3K signaling pathways has been suggested (5, 21–23). Dysfunction of these pathways is also linked to various diseases such as cardiovascular, renal, pulmonary, allergic, skeletal, and oral disorders. Mutations in the *WDR26* gene can also affect the assembly of the CTLH E3 complex, which is involved in maintaining cellular homeostasis through ubiquitination and proteasomal degradation, and is also involved in cell growth, proliferation and differentiation (14).

This study has several limitations that should be acknowledged. Firstly, the sample size is limited to a single case, which restricts the generalizability of our findings. Secondly, functional assays were not performed to validate the impact of the *WDR26* variant on cellular mechanisms. Consequently, our findings should be interpreted as preliminary observations. Future investigations involving larger cohorts of patients are warranted to refine the phenotypic spectrum, elucidate genotype–phenotype correlations, and identify additional phenotypic determinants.

Currently, there is no cure for Skraban-Deardorff syndrome caused by *WDR26* gene variants. Clinical management primarily relies on multidisciplinary comprehensive assessment and individualized symptomatic supportive care. This approach begins with a thorough clinical evaluation of the patient to determine the extent of disease involvement, covering neurodevelopment, intelligence, language, motor function, and the skeletal system. The core of treatment lies in targeted interventions: for neurodevelopmental delays and cognitive impairments, it is crucial to initiate long-term, individualized rehabilitation training as early as possible. This includes physical therapy, occupational therapy, speech therapy, and cognitive training to improve overall function. If seizures occur, appropriate anti-epileptic medications should be selected under the guidance of a neurologist, based on the seizure type and electroencephalogram (EEG) results. Furthermore, regular follow-ups should be conducted according to the severity of the condition to dynamically monitor developmental levels, seizure control, and other factors, allowing for timely adjustments to the treatment plan. For families with plans for future pregnancies, genetic counseling should be provided to explain the recurrence risk and discuss options such as prenatal diagnosis or preimplantation genetic testing. Simultaneously, psychological and social support should be offered to families to help them cope with the pressures of long-term care.

## 6 Conclusions

We describe a Chinese case of Skraban-Deardorff syndrome with a novel *WDR26* variant, along with the detailed clinical information, including growth and development disorders. The patients reported in literature exhibited various clinical features, such as intellectual disabilities, developmental delays, hypotonia, structural abnormalities in the central nervous system, and facial features that included a depressed nasal root, full nasal tip, abnormal gums, and wide spaces between the teeth. Furthermore, we provide a summary of the clinical characteristics observed in 37 documented cases with Skraban-Deardorff syndrome. Early-onset seizures were not detected in any of the reported Chinese cases, which is considered as the representative phenotype of Skraban-Deardorff syndrome. Since all reported patients had delayed speech, delayed language development should be considered a core symptom of Skraban-Deardorff syndrome. Unlike epilepsy, delayed language development is not manageable through medication. Therefore, it is necessary to evaluate more clinical phenotypes to ensure better diagnosis and earlier intervention. Since our patient is young, long-term follow-up is necessary to assess the progression of the disease in this case.

## Data Availability

The data presented in this study are deposited in the GSA for human repository, accession number SubHRA004486.
